# Supplementary material for: YdbL directly modulates YdbH-YnbE bridge formation to maintain Escherichia coli outer membrane homeostasis
Source: mBio. 2026 Jun 29;17(8):e00700-26. doi: 10.1128/mbio.00700-26 (PMC13464000; doi:10.1128/mbio.00700-26)
Supplement: Supplementary file 1 [file mbio.00700-26-s0001.pdf]

YdbL directly modulates YdbH-YnbE bridge formation to maintain *Escherichia coli* outer membrane homeostasis

Clare De'Ath[1†], Sujeet Kumar[2†], Sachi Chhibber[2], Benjamin F. Cooper[1], Emilia K. Taylor[1,3], Emily Jones[1], Thomas Lanyon-Hogg[3], Jani R. Bolla[4], Christina Redfield[4], Natividad Ruiz[2*] & Georgia L. Isom[1*]

[1] Sir William Dunn School of Pathology, University of Oxford, Oxford, OX1 3RE, UK

[2] Department of Microbiology, The Ohio State University, Columbus, OH 43210, USA

[3] Department of Pharmacology, University of Oxford, Oxford, OX1 3QT, UK

[4] Department of Biochemistry, University of Oxford, Oxford, OX1 3QU, UK

[†] Both authors contributed equally to this work

* Corresponding authors: Natividad Ruiz and Georgia L. Isom

Email: ruiz.82@osu.edu; georgia.isom@path.ox.ac.uk

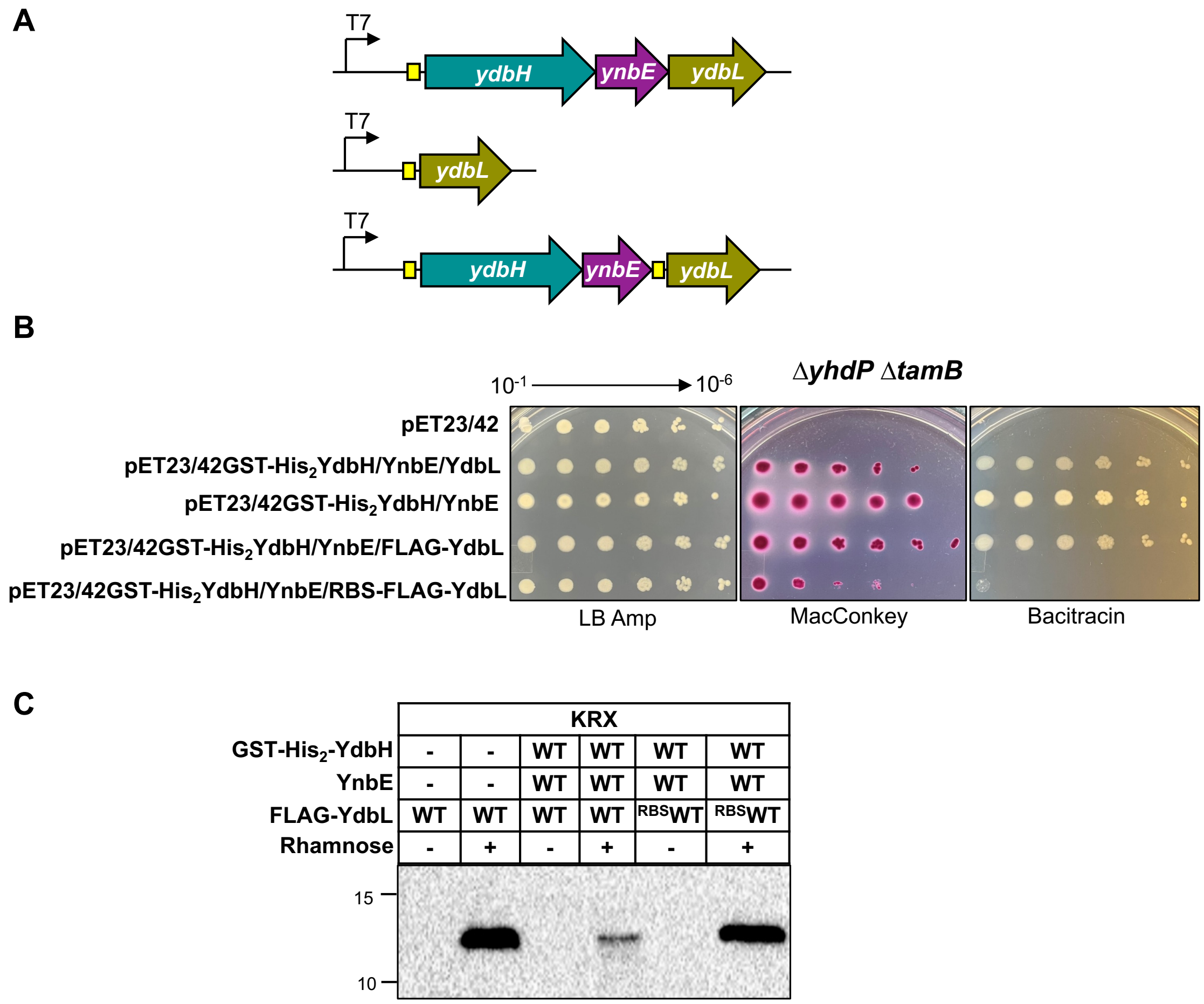

Figure S1 - In the absence of YhdP and TamB, YdbL is required for suppression of mucoidy by plasmid-encoded YdbH-YnbE and causes lethality in excess. (A) The genetic arrangement of T7 promoter, the ribosome binding site (RBS; yellow box), and *ydbH* (teal), *ynbE* (plum) and *ydbL* (gold) encoded on pET23/42-derived plasmids. **(B)** The effect of higher levels of YdbL on the growth of Δ*yhdP* Δ*tamB* double mutant was tested by spotting serially diluted cultures of Δ*yhdP* Δ*tamB* mutant strains carrying pET23/42-derived plasmids on LB agar containing ampicillin, MacConkey agar, and LB agar containing bacitracin. Increasing production of YdbL through introduction of an RBS upstream of the *ydbL* start codon on a pET23/42-derived plasmid impaired growth of the Δ*yhdP* Δ*tamB* double mutant on MacConkey agar and LB agar with bacitracin. **(C)** Immunoblot analysis showed that introducing an RBS upstream of *ydbL* on pET23/42-derived plasmids increased the cellular level of YdbL. KRX strains carrying FLAG-YdbL encoded on pET23/42-derived plasmid were grown to log phase, after which they were either left uninduced or induced with 0.2% rhamnose. They were then incubated for an additional 3 hours at 37 ºC before sample preparation. Whole-cell lysate samples were prepared as described in the Materials and Methods section, and proteins were separated on 15% SDS-polyacrylamide gel and probed with α-FLAG antibodies by immunoblotting. Molecular mass markers (in kDa) are shown on the left. All data are representative of three independent experiments.

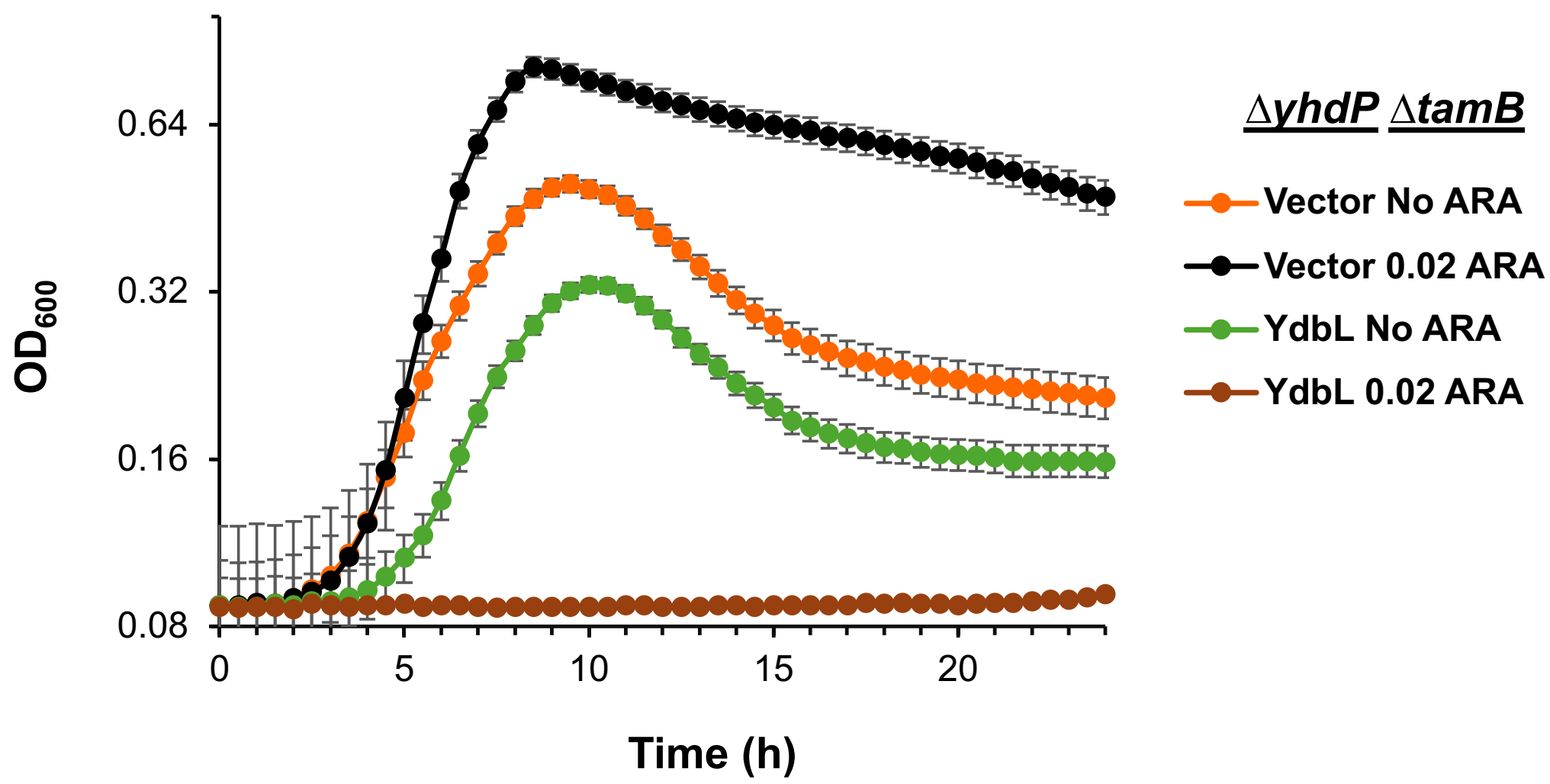

Figure S2 - Overexpression of YdbL from pBAD33 causes lethality in the Δ*yhdP::frt* Δ*tamB::frt* mutant. Growth curves of cultures grown in LB containing spectinomycin with or without 0.02% arabinose at 37 °C. The metabolic effect of arabinose consumption allowed the Δ*yhdP* Δ*tamB* double mutant carrying the pBAD33-SpecR vector to grow better under 0.02% arabinose condition than in its absence. The *ΔyhdP ΔtamB* double mutant carrying pBAD33-SpecR-RBS-YdbL failed to grow in the presence of 0.02% arabinose, and the lysis of this strain compared to *ΔyhdP ΔtamB* double mutant carrying pBAD33-SpecR vector was faster even without arabinose induction due to leaky expression of *ydbL*. OD_{600} values are represented on a logarithmic scale. Data represent the average and standard deviation from three biological replicates.

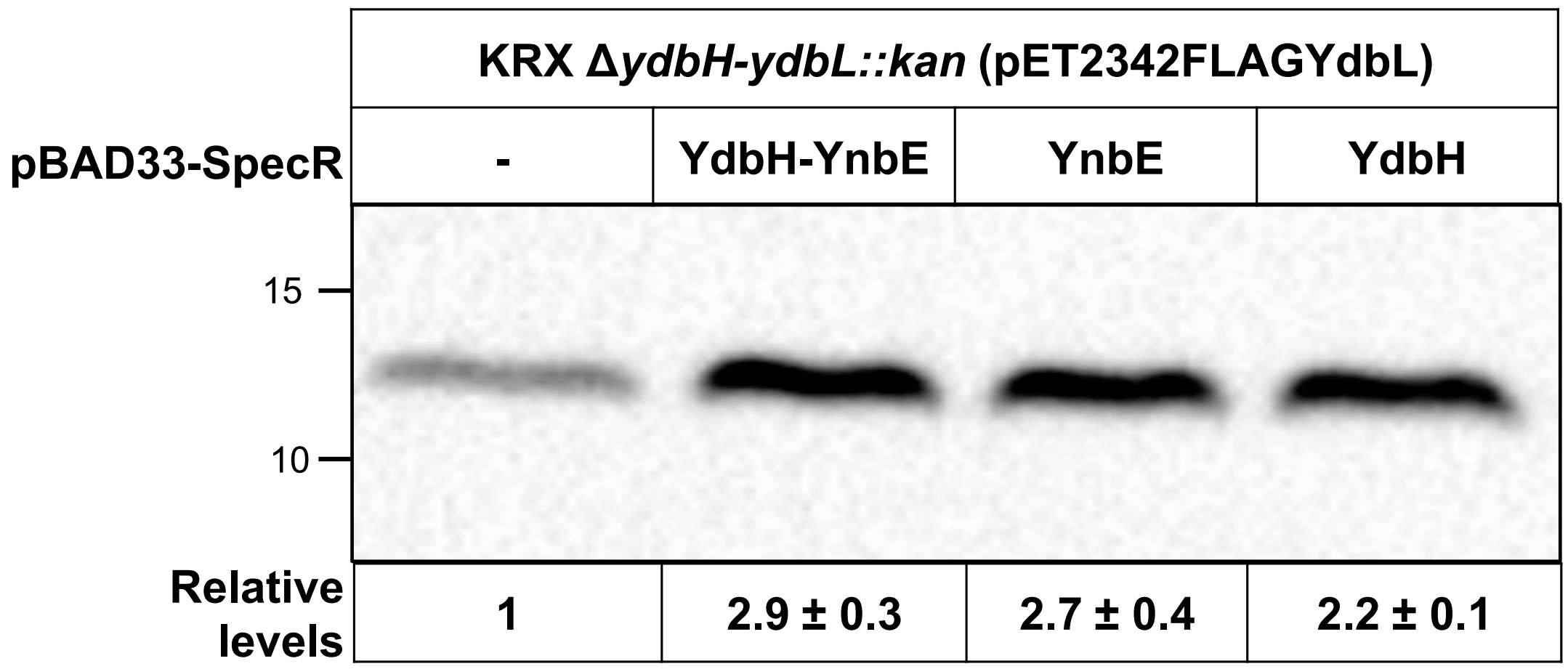

Figure S3 - Producing YdbH and/or YnbE *in trans* increases the levels of YdbL. KRX Δ*ydbH-ydbL*::*kan* strains carrying pET23/42FLAG-YdbL and pBAD33-SpecR-derived plasmids were grown to log phase. Production of YdbH and/or YnbE was induced for three hours at 37 ºC with 0.2% rhamnose. Whole-cell lysate samples were prepared as described in Materials and Methods, and proteins were separated on 15% SDS-polyacrylamide gel and probed with α-FLAG antibodies by immunoblotting. When YdbH, YnbE, or both were produced from the pBAD33 plasmid, increased levels of FLAG-YdbL produced from the pET23/42 plasmid were detected. For calculating relative levels, the signal from samples derived from cells with a pBAD33 vector plasmid was set to 1. Molecular mass markers (in kDa) are shown on the left. All data are representative of three independent experiments.

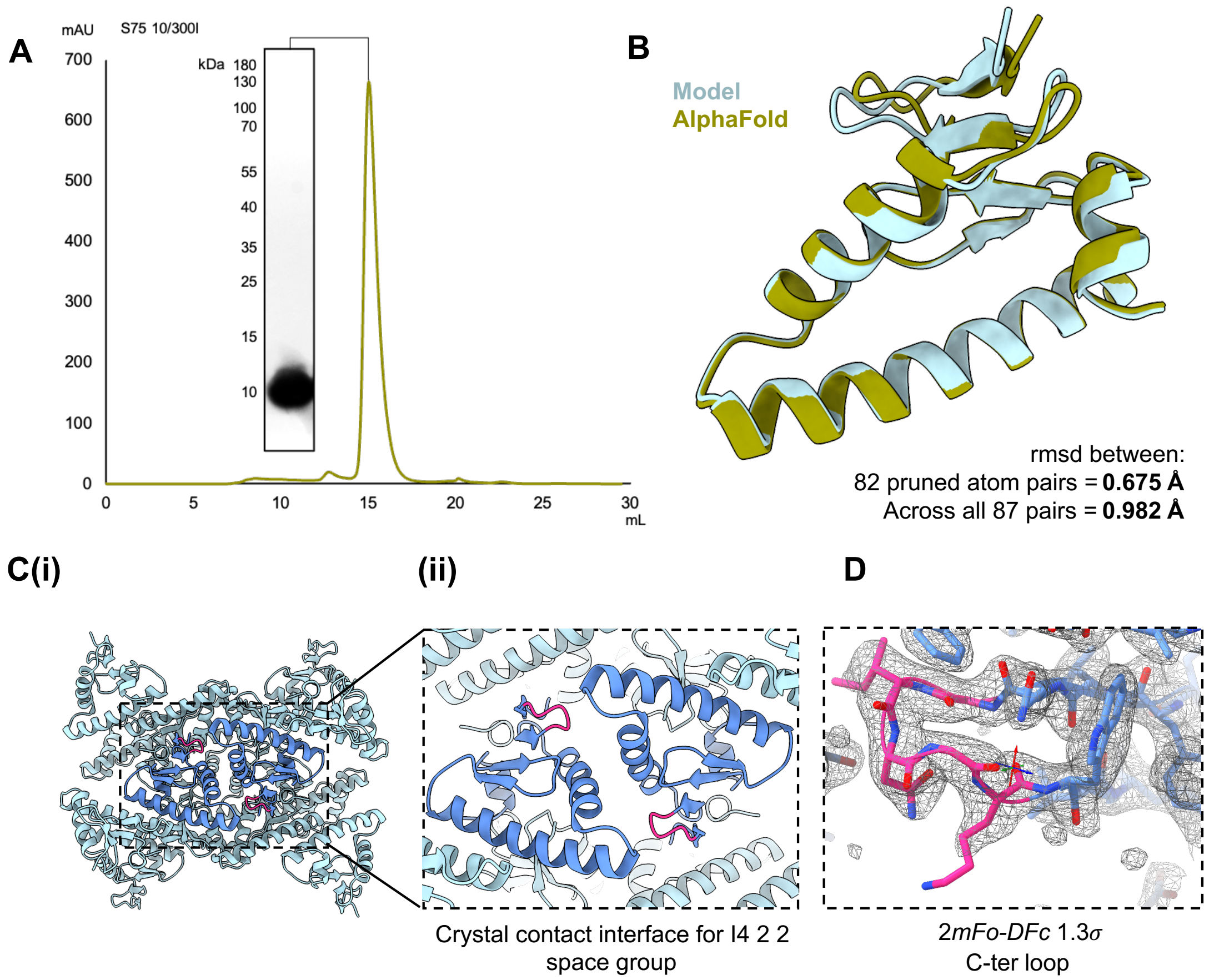

Figure S4 - X-ray crystallography (XRC) YdbL model is concurrent with AlphaFold. a. Purification of YdbL for XRC. S75 10/300I SEC trace reveals a homogenous protein peak. Coomassie-stained SDS-PAGE shows full-length protein without any major contaminants or signs of degradation/aggregation. **(B)** Overlay of AlphaFold2-YdbL (P76076) with 2.12 Å model reveals highly comparable secondary structure with an rmsd score below 1.0 Å across all atom pairs. **(C) (i)** YdbL crystal contact interface with I4 2 2 space group for YdbL crystal lattice shown in sky blue with two representative YdbL monomers highlighted in cornflower blue. **(ii)** Magnified region showing the crystal-contact interfaces as corroborated by PDBePISA reveals the C-terminal loop (magenta) may be mobile and subject to an alternative conformation to the AlphaFold prediction in this packing lattice. **(D)** The C-terminal loop is well represented by the electron density map for modelling in this conformation, with colors matching what is used in **(C)**.

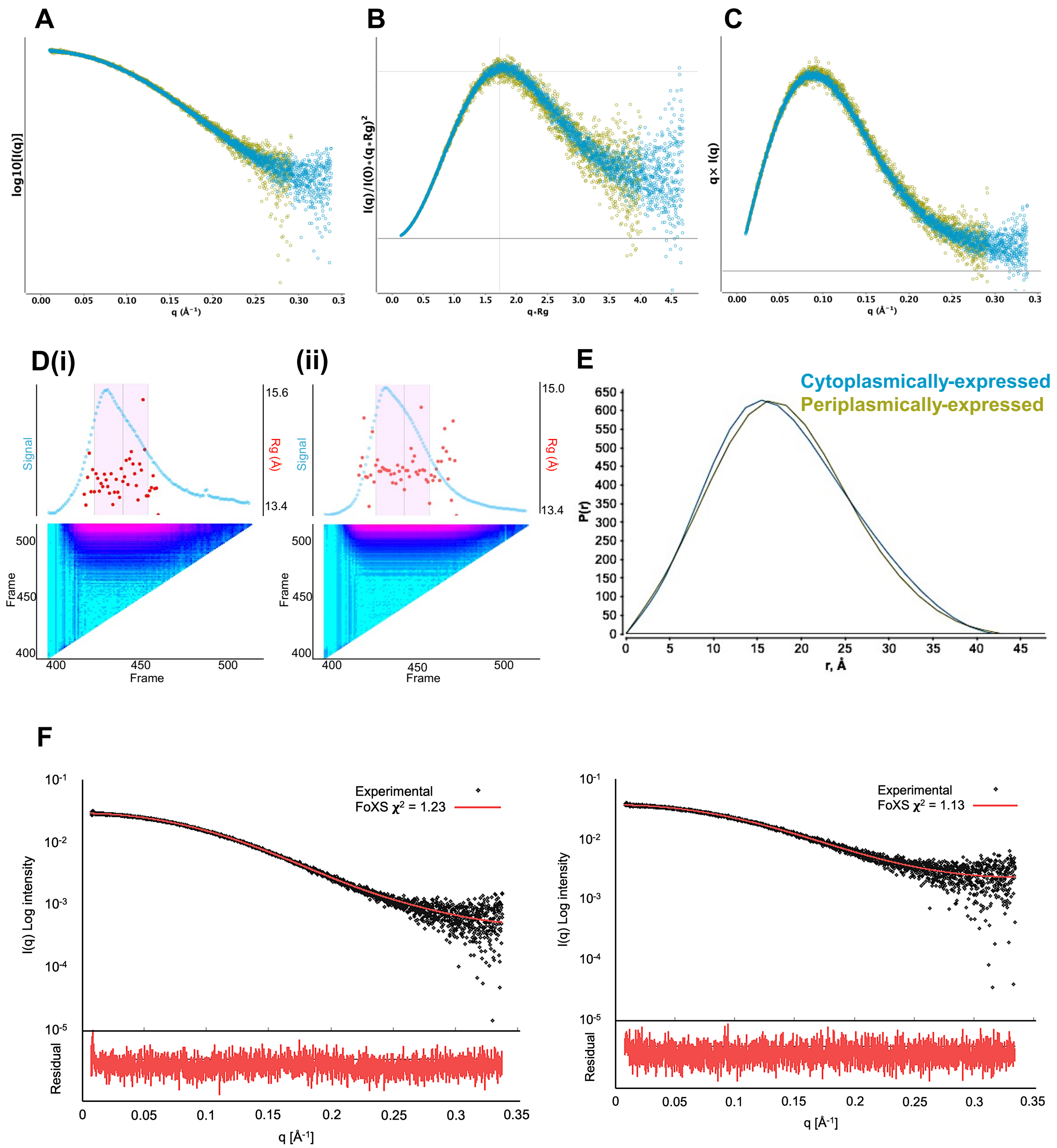

Expression	χ^2	c1, c2	Rg	#atoms
Cytoplasm	1.23	1.02, 0.52	13.24	682
Periplasm	1.13	1.05, -0.18	13.24	682

Figure S5 - XRC structure matches solution structure via SEC-SAXS. a. Log10 SAXS intensity versus scattering vector, *q*. Plotted range represents the positive only data within the specified *q*-range. **(B)** Dimensionless Kratky plot. Cross-hair marks the Guinier-Kratky point (1.732, 1.1), the main peak position for globular particles. **(C)** Total scattered intensity plot. Plot readily demonstrates negative intensities at high-*q*. Over-subtraction of background leads to significant negative intensities. Likewise, under-subtraction can be observed as an elevated baseline at high-*q*. The horizontal line is drawn at y=0. **(D)** SEC signal intensity plots for **(i)** cytoplasmically-expressed and **(ii)** periplasmically-expressed YdbL depict how Rg varies across frames, with the

frame-by-frame panel below depicting their relative agreement, whereby cyan indicates high similarity and pink indicates low similarity. **(E)** The plotted P(r) model function. Cytoplasmically- and periplasmically-expressed constructs are represented in blue and gold respectively. **(F)** SEC-SAXS experimental data was analysed in comparison to the refined .pdb model using the FoXS server with cytoplasmically- and periplasmically-expressed data fitted to the crystal structure from left to right. Statistical fit of Chi-squared (χ^2) and the free parameters - c1 (scaling of atomic radius) within limits of $0.99 \leq c_1 \leq 1.05$ and c2 (adjustment of hydration layer and bulk water densities) within limits of $-2.0 \leq c_2 \leq 4.0$ are also shown below for both experimental datasets. Rg and number of atoms in the crystal structure are also recorded.

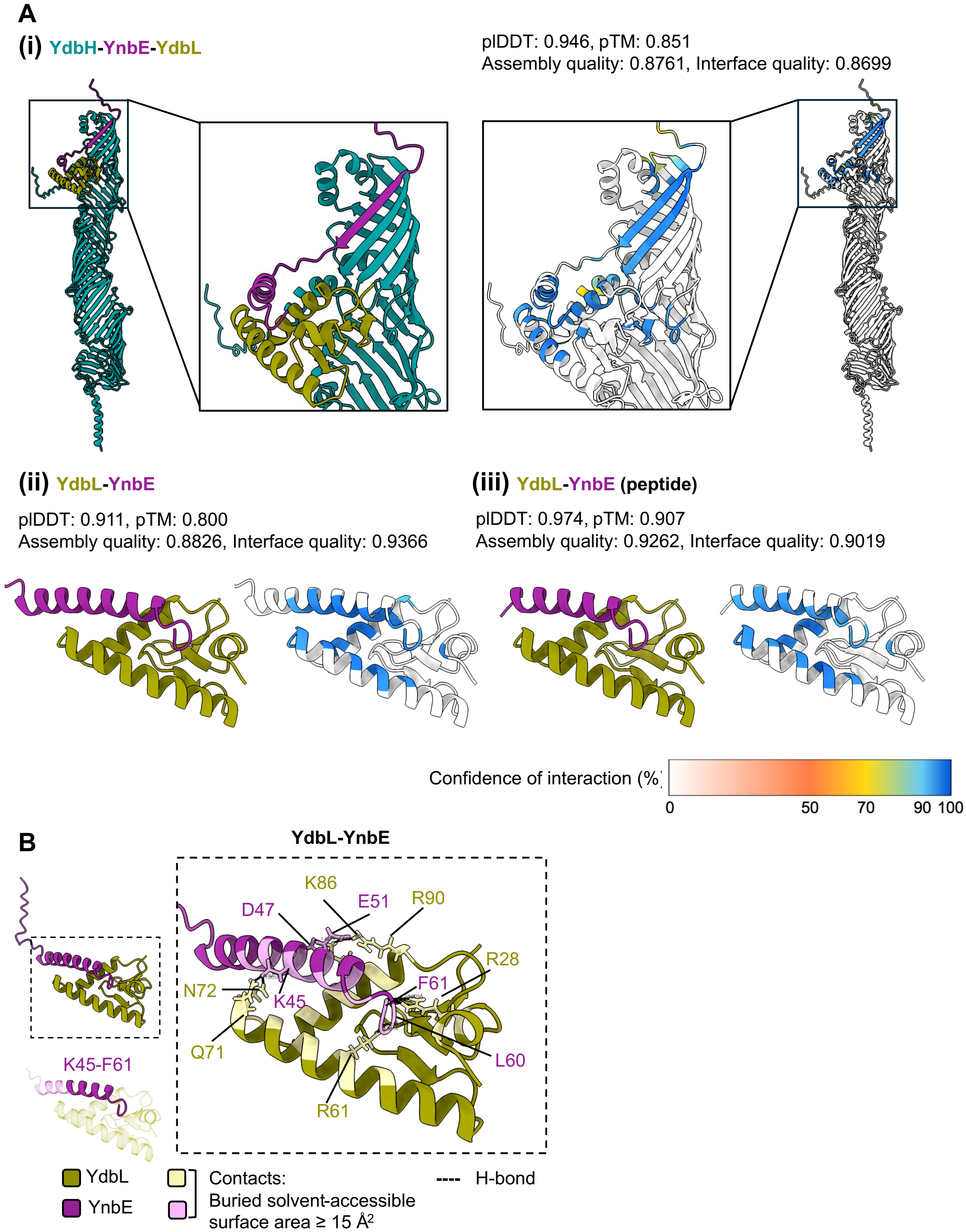

Figure S6 - MultiFold2- and ModFolddock2-predicted interaction interfaces between YdbH, YnbE and YdbL. (A) Confidence of MultiFold2-predicted interaction interfaces by ModFolddock2 for **(i)** YdbH-YnbE-YdbL, **(ii)** YdbL-YnbE and **(iii)** YdbL-YnbE peptide. For each complex, the left-hand model is colored by chain (YdbH in teal, YnbE in plum, YdbL in gold). The right-hand model shows the confidence of interaction (%). Colored residues are predicted to form the interaction interfaces between proteins. Their color is scaled by the confidence of interaction with 90-100%

being highly likely versus lower confidence predictions around 50%. **(B)** Predicted MultiFold2 interface for YdbL-YnbE colored by protein (YdbL in gold, YnbE in plum). A magnified view of the interaction interface reveals the contact region and predicted hydrogen bonds. Residues predicted to mediate direct interactions are labelled.

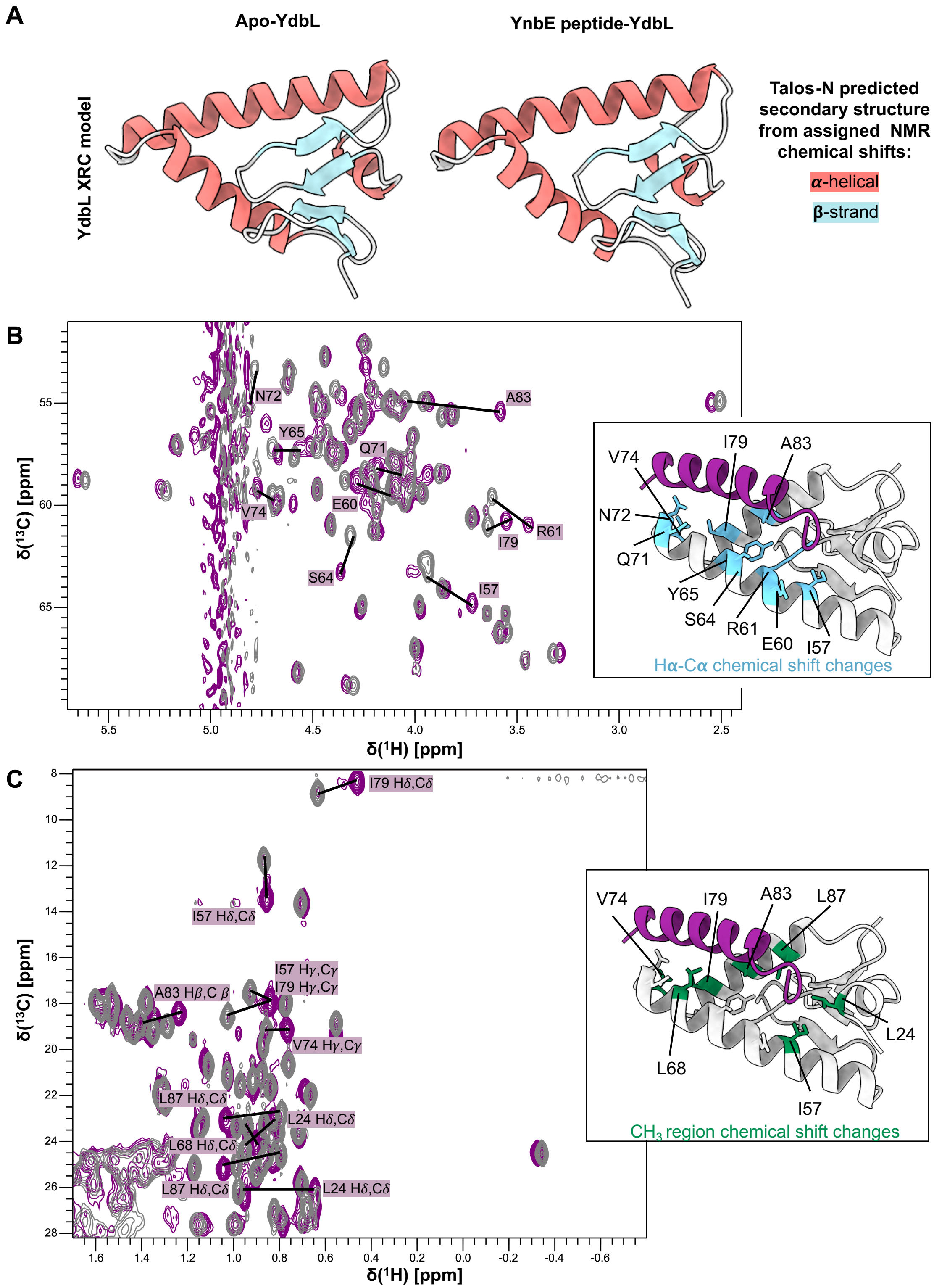

Figure S7 - Analysis of secondary structure and chemical shift changes between apo- and peptide-bound YdbL did not reveal any significant conformational changes. a. Talos-N secondary structure prediction for apo- (left) vs peptide-bound (right) YdbL mapped onto the crystal model of YdbL. Red depicts **α**-helical structure, blue depicts **β**-strand structure and white depicts

loops or unstructured regions. Residues are identified as **α**-helical or **β**-strand if the Talos-N analysis of backbone chemical shifts assigns a helix or strand probability of >0.5, respectively. **(B)** Overlaid 2D ^{1}H-^{13}C HSQC spectra showing the H**α**-C**α** region for apo- (grey) and peptide-bound (purple) YdbL. The H**α**-C**α** peaks that are labelled show some of the most significant chemical shift changes that occur upon peptide binding. Most of these residues also show large amide shift changes in Figure 3(C). Interestingly, most of the residues highlighted in this spectrum show an increase in the C**α** chemical shift upon peptide binding. The YdbL-YnbE peptide MultiFold2 model shown to the right of the spectra is labelled with residues in blue indicating chemical shift differences for H**α**-C**α** at 0.1 ppm or more. Since they are located in helical regions, this increase in C**α** chemical shift may indicate a stabilization of the helical structure rather than a significant conformational change; this observation is in agreement with the MultiFold2 predictions (Figure 2(D)). **(C)** Overlaid 2D ^{1}H-^{13}C HSQC spectra showing the methyl (CH_3) region for apo- (grey) and peptide-bound (purple) YdbL. Labelled peaks focus on the most significant chemical shift changes. As shown by the model to the right of the spectra, the methyl groups showing large chemical shift changes are all located in the identified peptide binding site (colored green), with many of these residues also showing the most significant amide shift changes shown in Figure 3(C). The observed changes in methyl group chemical shifts are likely to arise from small side chain conformational rearrangement due to direct contact between these groups and residues in the YnbE peptide and/or from movements of nearby aromatic amino acid side chains which lead to through-space changes in chemical shifts.

A

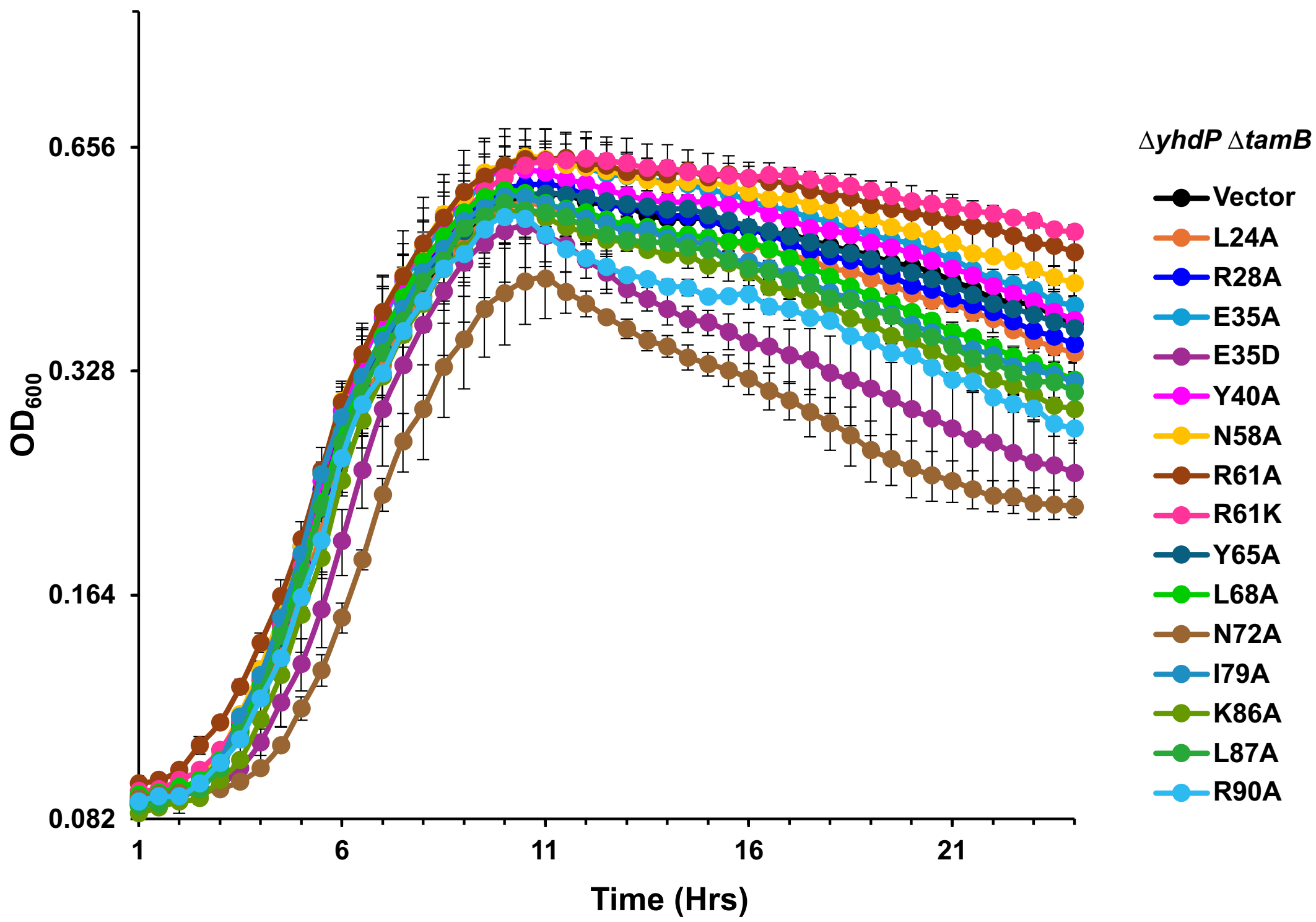

B

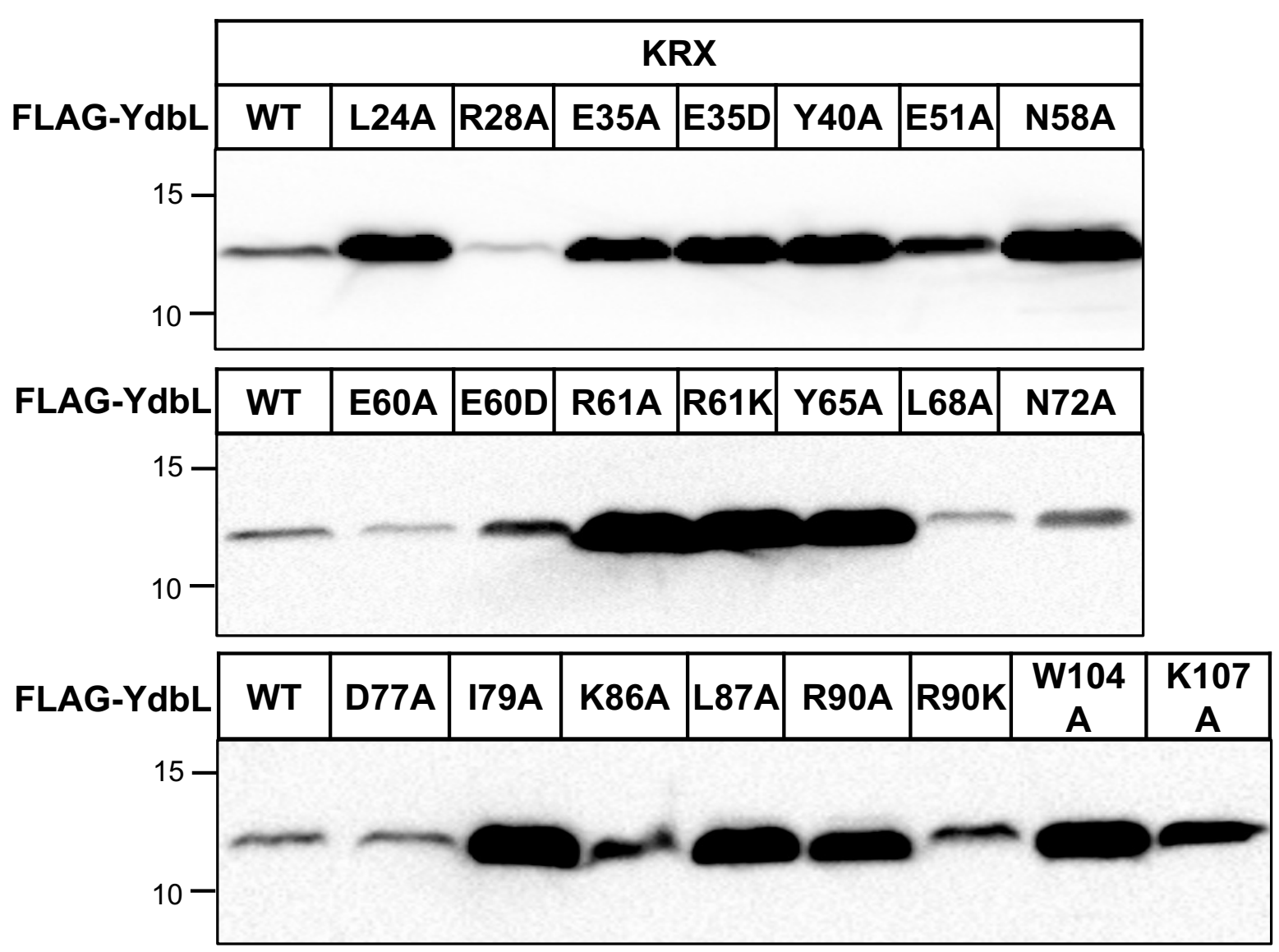

Figure S8 - Growth curves and immunoblots of YdbL variants a. Growth curves for non-functional YdbL variants. Overnight cultures of the Δ*tamB* Δ*yhdP* double mutant carrying FLAG-YdbL variants encoded on pET23/42 plasmid were diluted (1:1000) in LB containing ampicillin and grown at 37°C for 24 hrs. OD_{600} values are represented on a logarithmic scale. Data represent the average and standard deviation from three biological replicates. **(B)** Immunoblots showing the cellular levels of YdbL variants. KRX strains carrying FLAG-YdbL variants encoded on the pET23/42-derived plasmid were grown to log phase and then for three additional hours at 37 ºC after induction with 0.2% rhamnose. Whole-cell lysate samples were prepared as described in the

Materials and Methods section, and proteins were separated on 15% SDS-polyacrylamide gel and probed with α-FLAG antibodies by immunoblotting. Most, but not all, non-functional YdbL variants showed increased levels compared to wild-type YdbL (WT). Molecular mass markers (in kDa) are shown on the left. All data are representative of three independent experiments.

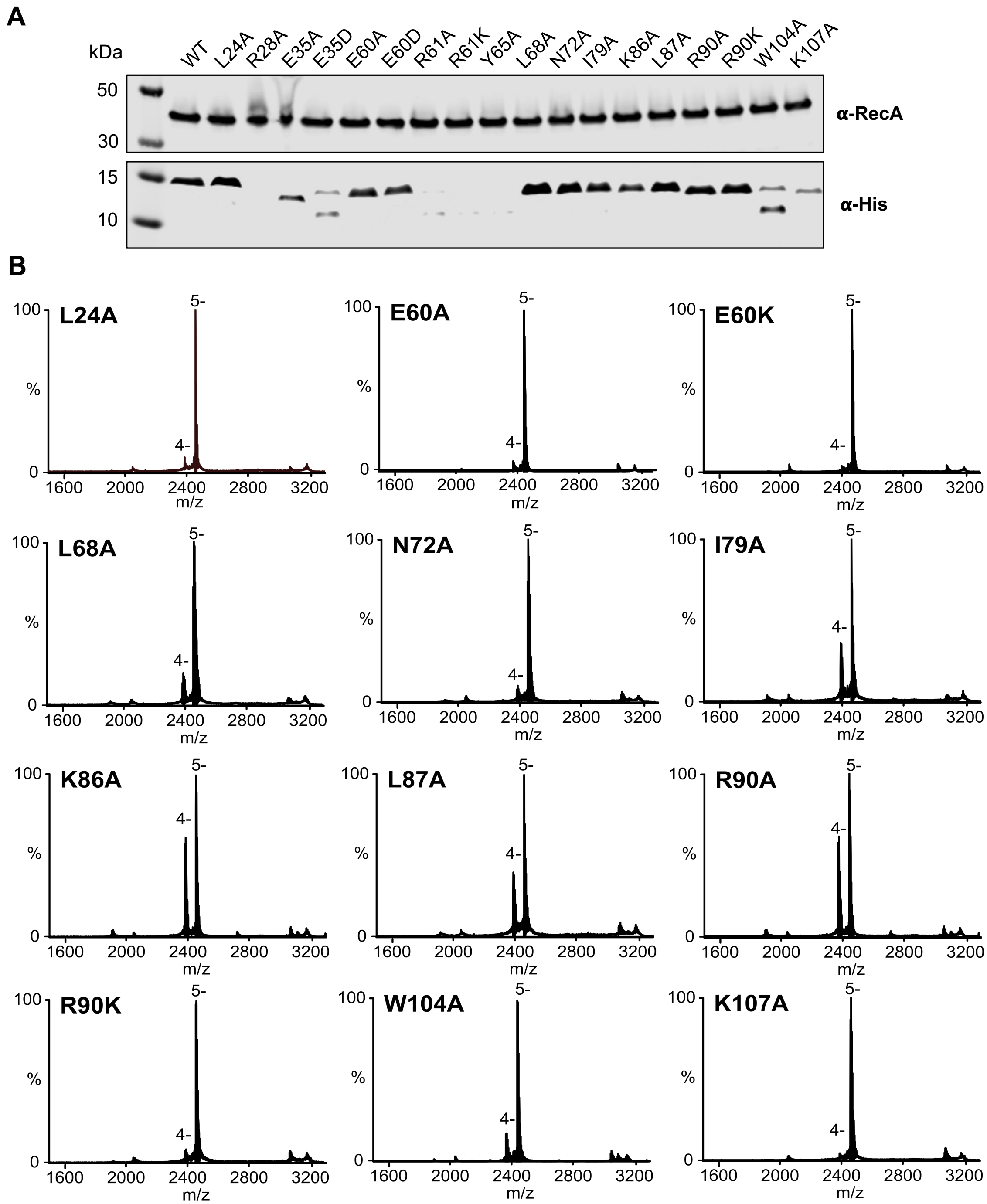

Figure S9 - nMS data for all mutants. a. Expression test for all YdbL variant proteins with substitutions at the YnbE-binding interface. Recombinantly expressed protein variants were analyzed via immunoblot against His-YdbL (α-His) from whole- cell lysates. An α-RecA immunoblot was included for a loading control. The expression test is representative across three biological replicates. **(B)** Native mass spectra of each mutant in the presence of 3.5 µM peptide. The highest intensity charge state series are labelled: apo (4-) and peptide-bound (5-) Spectra are representative across three technical replicates.

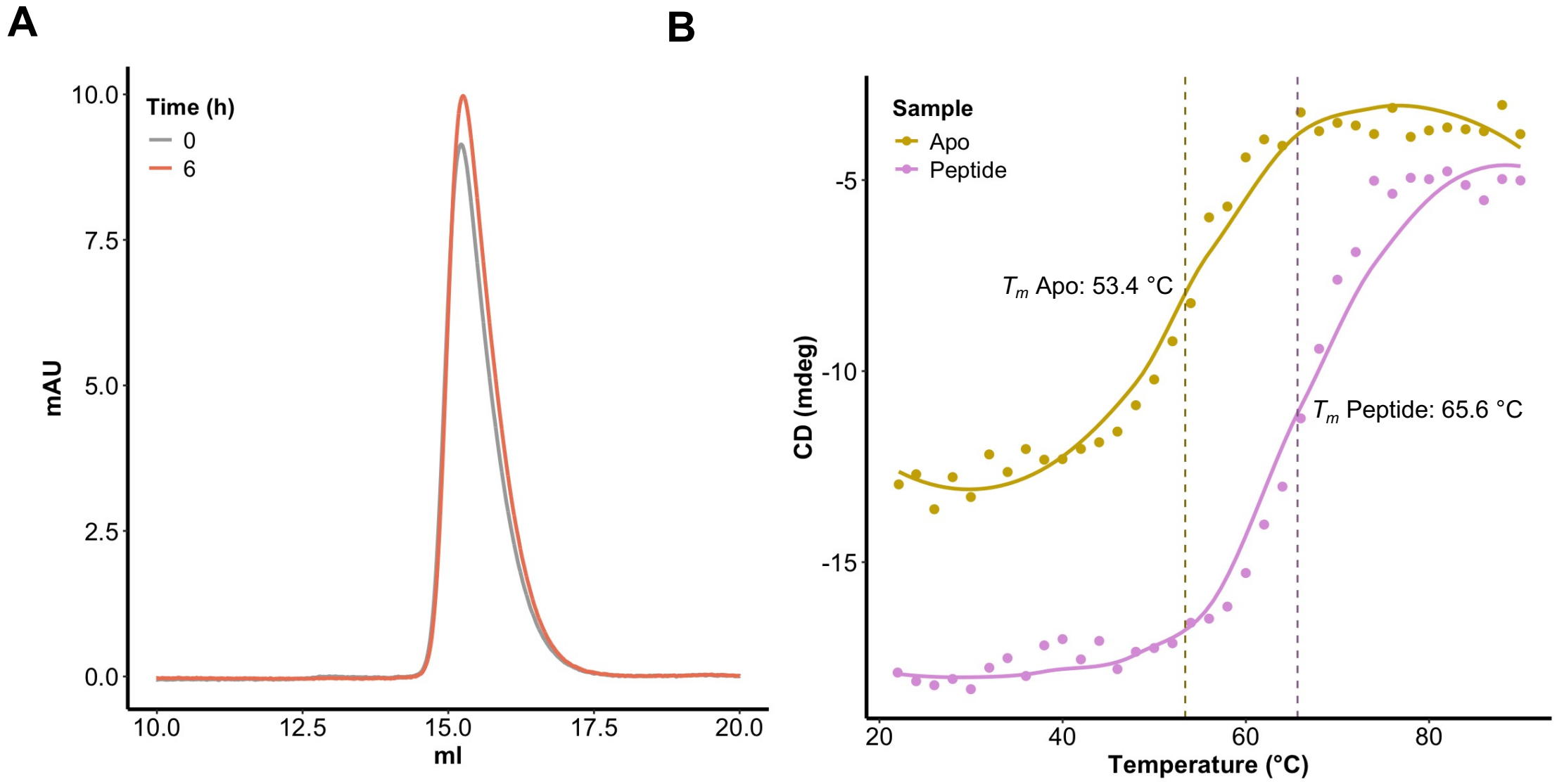

Figure S10 - Biophysics for YdbL reveals it is thermostable, folded and does not aggregate in the DegP degradation assay conditions. a. SEC aggregation assay. YdbL was incubated at 37 °C for 0 and 6 hours in standard assay buffer before loading onto an S75 10/300**l** column. At both timepoints, YdbL eluted as a monodisperse peak with no visible aggregate peak or loss of folded sample. **(B)** Thermal denaturation analysis shows peptide-bound YdbL has a higher thermal stability than apo-YdbL. The CD melt curves for apo-YdbL (gold) and peptide-bound YdbL (purple) are plotted as the circular dichroism millidegrees against temperature. A wavelength of 222 nm was selected to monitor loss of α-helical secondary structure with dots used to show raw data with scans from 22-90 °C in 2 °C step intervals. The solid lines depict the smoothened melting curve. The midpoint of sharp melting transition occurs at 53.4 °C for apo-YdbL and 65.6 °C for peptide-bound YdbL.

Table S1 - Strains used in this study

Strain	Genotype	Source
DH5α	F$^-$ φ80*lacZ*ΔM15 Δ(*lacZYA-argF*)U169 *recA1 endA1 hsdR17* (r$_K^-$, m$_K^+$) *phoA sup*E44 λ$^-$ *thi*-1	Life Technologies
T7 Express	*fhuA2 lacZ::T7 gene1 [lon] ompT gal sulA11 R(mcr-73::miniTn10*--Tet^S*)2 [dcm] R(zgb-210::Tn10*--Tet^S*) endA1 Δ(mcrC-mrr)114::IS10*	NEB
MG1655	Wild type	(1)
NR5161	Δ*yhdP*::*frt* Δ*tamB*::*frt*	(2)
KRX	[F´, traD36, Δ*ompP*, *proA+B+, lacIq*, Δ(*lacZ)*M15] Δ*ompT*, *endA1*, *recA1, gyrA96 (Nalr), thi-1, hsdR17 (rk–, mk+), e14– (McrA–), relA1, supE44*, Δ(*lac-proAB*), Δ(*rhaBAD*)::T7 RNA polymerase.	Promega
NR5530	KRX (pKD46)	This study
NR6436	MG1655 Δ*ydbH-ydbL::kan*	(3)
NR6658	Δ*yhdP*::*frt* Δ*tamB*::*frt* (pET23/42)	(3)
NR7056	NR5161 (pET23/42GST-His$_2$-YdbH/YnbE/YdbL)	(3)
NR7254	NR5161 (pET23/42GST-His$_2$-YdbH)	(3)
NR7258	MG1655 (pET23/42GST-His$_2$-YdbH/ΔYnbE/YdbL)	(3)
NR7352	NR5161 (pET2342 GST-His$_2$-YdbH /YnbE/FLAG-YdbL)	(3)
NR7359	MG1655 Δ*ydbH-ydbL::kan* (pSup-BpaRS-6TRN)	(3)
NR7817	NR5161 (pET23/42 pET23/42GST-His$_2$-YdbH/YnbE-FLAG$_{STOP}$)	(3)
NR7818	NNR5161 (pET23/42YnbE-FLAG$_{STOP}$)	(3)
NR7859	KRX (pET23/42FLAGYdbL)	This study
NR7867	KRX (pET23/42GST-His$_2$-YdbH /YnbE/FLAG-YdbL)	This study
NR7868	MG1655 (pET23/42YdbL)	(3)
NR7885	MG1655 Δ*yhdP::frt* (pET23/42YdbL)	(3)
NR7890	MG1655 Δ*tamB::frt* (pET23/42YdbL)	(3)
NR7902	NR5161 (pET23/42GST-His$_2$-YdbH/YnbE$_{STOP}$)	(3)
NR7980	KRX Δ*ydbH-ydbL::kan*	This study
NR8194	KRX (pET23/42FLAG/YdbL/E35A)	This study
NR8195	KRX (pET23/42FLAG/YdbL/R61A)	This study
NR8196	KRX (pET23/42FLAG/YdbL/Y65A)	This study
NR8197	KRX (pET23/42FLAG/YdbL/W104A)	This study
NR8198	KRX (pET23/42FLAG/YdbL/K107A)	This study
NR8202	NR5161 (pET23/42FLAG/YdbL/E35A)	This study
NR8203	NR5161 (pET23/42FLAG/YdbL/R61A)	This study
NR8204	NR5161 (pET23/42FLAG/YdbL/Y65A)	This study
NR8212	NR5161 (pET23/42FLAG/YdbL/R28A)	This study
NR8213	NR5161 (pET23/42FLAG/YdbL/Y40A)	This study
NR8214	KRX (pET23/42FLAG/YdbL/R28A)	This study
NR8215	KRX (pET23/42FLAG/YdbL/Y40A)	This study
NR8224	NR5161 (pBAD33SpecR)	This study
NR8225	NR5161 (pBAD33-SpecR-NativeYdbH/YnbE/YdbL)	This study
NR8226	NR5161 [pBAD33-SpecR-RBS-YdbH/YnbE/YdbL]	This study
NR8227	NR5161 [pBAD33-SpecR-RBS-YdbL]	This study
NR8278	NR5161 [pBAD33SpecR-RBS-YdbL;pET23/42]	This study
NR8279	NR5161 [pBAD33SpecR-RBS-YdbL; pET23/42GST-His$_2$-YdbH/YnbE/YdbL]	This study
NR8280	NR5161 [pBAD33SpecR-RBS-YdbL; pET23/42GST-His$_2$-YdbH/YnbE-FLAG/YdbL]	This study
NR8281	NR5161 [pBAD33SpecR-RBS-YdbL; pET23/42GST-His$_2$-YdbH/YnbE$_{STOP}$]	This study
NR8282	NR5161 [pBAD33SpecR-RBS-YdbL; pET23/42GST-His$_2$-YdbH/YnbEFLAG$_{STOP}$]	This study

NR8283	NR5161 [pBAD33SpecR-RBS-YdbL; pET23/42GST-His_2-YdbH]	This study
NR8284	NR5161 [pBAD33SpecR-RBS-YdbL; pET23/42YnbEFLAG$_{STOP}$]	This study
NR8335	NR5161 [pBAD33SpecR-RBS-YdbL; pET23/42YnbE$_{STOP}$]	This study
NR8357	MG1655 Δ*ydbH*-Δ*ydbL::kan* [pSUP-BPARS-6TRN; GST-His_2-YdbH/YnbE-FLAG(K27Am)$_{STOP}$; pBAD33-SpecR)]	This study
NR8358	MG1655 Δ*ydbH*-Δ*ydbL::kan* [pSUP-BPARS-6TRN; GST-His_2-YdbH/YnbE(K27Am)-FLAG$_{STOP}$; pBAD33-SpecR-RBS-YdbL]	This study
NR8359	MG1655 ΔydbH-ydbL::kan [pSUP-BPARS-6TRN; GST-His_2-YdbH /YnbE(D47Am)-FLAG$_{STOP}$;pBAD33-SpecR]	This study
NR8360	MG1655 ΔydbH-ydbL::kan [pSUP-BPARS-6TRN; GST-His_2-YdbH /YnbE(D47Am)-FLAG$_{STOP}$; pBAD33-SpecR-RBS-YdbL]	This study
NR8383	KRX (pET23/42FLAG-YdbL/R61K)	This study
NR8384	KRX (pET23/42FLAG-YdbL/N58A)	This study
NR8385	KRX (pET23/42FLAG-YdbL/E35D)	This study
NR8391	MG1655 Δ*ydbH-ydbL::kan* [pSUP-BPARS-6TRN; GST-His_2-YdbH /YnbE(E41Am)-FLAG$_{STOP}$; pBAD33SpecR]	This study
NR8392	MG1655 Δ*ydbH-ydbL::kan* [pSUP-BPARS-6TRN; pET23/42 GST-His_2-YdbH /YnbE(E41Am)-FLAG$_{STOP}$; pBAD33SpecR-RBS-YdbL]	This study
NR8395	MG1655 ΔydbH-ydbL::kan [pSUP-BPARS-6TRN; GST-His_2-YdbH /YnbE(F61Am)-FLAG$_{STOP}$; pBAD33SpecR]	This study
NR8396	MG1655 ΔydbH-ydbL::kan [pSUP-BPARS-6TRN; pET23/42GST-His_2-YdbH /YnbE(F61Am)-FLAG$_{STOP}$; pBAD33SpecR-RBS-YdbL]	This study
NR8455	KRX Δ*ydbH-ydbL::kan* (pET23/42FLAG-YdbL)	This study
NR8456	KRX Δ*ydbH-ydbL::kan* (pET23/42GST-His_2-YdbH/YnbE/FLAG-YdbL)	This study
NR8460	NR5161 (pET23/42FLAG-YdbL/E35D)	This study
NR8461	NR5161 (pET23/42FLAG-YdbL/N58A)	This study
NR8462	NR5161 (pET23/42FLAG-YdbL/R61K)	This study
NR8484	KRX Δ*ydbH-ydbL::kan* (pET23/42FLAG-YdbL; pBAD33SpecR)	This study
NR8485	KRX Δ*ydbH-ydbL::kan* [pET23/42FLAG-YdbL; pBAD33SpecRBS-YdbH/YnbE]	This study
NR8501	KRX (pET23/42FLAGYdbL/E51A)	This study
NR8502	KRX (pET23/42FLAGYdbL/N72A)	This study
NR8503	KRX (pET23/42FLAGYdbL/D77A)	This study
NR8504	KRX (pET23/42FLAGYdbL/K86A)	This study
NR8598	KRX Δ*ydbH-ydbL::kan* [pET23/42FLAG-YdbL; pBAD33Spec-RBS-YnbE]	This study
NR8609	KRX Δ*ydbH-ydbL::kan* [pET23/42FLAG-YdbL; pBAD33SpecRBS-YdbH]	This study
NR8642	NR5161 (pET23/42FLAG-YdbL/L68A)	This study
NR8643	NR5161 (pET23/42FLAG-YdbL/I79A)	This study
NR8644	NR5161 (pET23/42FLAG-YdbL/L87A)	This study
NR8645	NR5161 (pET23/42FLAG-YdbL/R90A)	This study
NR8650	KRX (pET23/42FLAG-YdbL/E60A)	This study
NR8651	KRX (pET23/42FLAG-YdbL/E60D)	This study
NR8652	KRX (pET23/42FLAG-YdbL/L68A)	This study
NR8653	KRX (pET23/42FLAG-YdbL/I79A)	This study
NR8654	KRX (pET23/42FLAG-YdbL/L87A)	This study
NR8655	KRX (pET23/42FLAG-YdbL/R90A)	This study
NR8656	KRX (pET23/42FLAG-YdbL/R90K)	This study
NR8673	NR5161 (pET23/42FLAG-YdbL/L24A)	This study
NR8674	NR5161 (pET23/42FLAG-YdbL/N72A)	This study
NR8675	NR5161 (pET23/42FLAG-YdbL/K86A)	This study

Table S2 - Primers used in the study.

Primer name	Sequence 5'-3'	Purpose
5YdbH deletion	ATGAAAATTTTACTGGCTGCGTTGACGTCATCTTTTATGC	SDM
3YnbE deletion	TCATTGTTTTTCCTCACACTCTTTTCCTTGCGGACAGT	SDM
3pET2342NdeIStart	CATATGTATATCTCCTTCTTAAAGTTAAACAAAATTATTTCTAGAG	Deletion of NdeI-BamHI region
5YdbH-YnbE deletion	AAGAAAACATTACTTCTTTGTGCGTTTCTTGTTGGGC	SDM
3YdbLdeletion	TCAGAAAAGATCGCTACGAGTTTCAAGCAGCTCTTCGAC	SDM
5YnbE K27Amber	CTGCACCTTAGGAACCGATCACTATCAATATGAACG	SDM
3YnbE K27Amber	GATCGGTTCCTAAGGTGCAGCGACTTCAATGCGAGGCGTAC	SDM
5YnbE E41Amber	TTGAGCATTAGATCATCATCAAGGCAGACAAAGATGTCG	SDM
3YnbE E41Amber	GATGATGATCTAATGCTCAATTTTAACGTTCATATTGATAG	SDM
5YnbE D47Amber	CAAGGCATAGAAAGATGTCGAAGAGCTGCTTGAAACTCG	SDM
3YnbE D47Amber	GACATCTTTCTATGCCTTGATGATGATCTCATGCTC	SDM
5YnbEF61AmberFLAG	GCGATCTTTAGGGAAGCGGAAGCGATTATAAAGATGACG	SDM
3YnbEF61AmberFLAG	CCGCTTCCCTAAAGATCGCTACGAGTTTCAAGCAGCTC	SDM
5YdbL NtermFLAG	GATTATAAAGATGACGATGACAAATTGACTCTGGATGAAGCCAGAACTCAGGG	Inserting N-terminal FLAG-tag in YdbL
3YdbL NtermFLAG	TGCCATTACATTGCTGCTTACCAGCCCAACAAGAAACGCAC	Inserting N-terminal FLAG-tag in YdbL
5YdbL L24A	AAATTGACTGCGGATGAAGCCAGAACTCAGGGGCGGGTAG	SDM
3YdbL L24A	GCTTCATCCGCAGTCAATTTGTCATCGTCATCTTTATAATC	SDM
5YdbL R28A	GATGAAGCCGCAACTCAGGGGCGGGTAGGTGAAACATTTTAC	SDM
3YdbL R28A	CCCCTGAGTTGCGGCTTCATCCAGAGTCAATTTGTCATC	SDM
5YdbL E35A	GGGTAGGTGCAACATTTTACGGTTATCTGGTTGCGCTG	SDM
3YdbL E35A	GGGTAGGTGCAACATTTTACGGTTATCTGGTTGCGCTG	SDM
5YdbL E35D	GGGTAGGTGACACATTTTACGGTTATCTGGTTGCGCTG	SDM
3YdbL E35D	GTAAAATGTGTCACCTACCCGCCCCTGAGTTCTGGCTTC	SDM
5YdbL Y40A	TTTTACGGTGCTCTGGTTGCGCTGAAAACGGATGCTG	SDM
3YdbL Y40A	GCAACCAGAGCACCGTAAAATGTTTCACCTACCCGCCCC	SDM
5YdbL E51A	CTGAAACAGCGAAATTAGTAGCCGACATTAATGCCGAAC	SDM
3YdbL E51A	TACTAATTTCGCTGTTTCAGCATCCGTTTTCAGCGCAAC	SDM

5YdbL N58A	CCGACATTGCAGCCGAACGTAAAGCGAGTTACCAAC	SDM
3YdbL N58A	CGTTCGGCTGCAATGTCGGCTACTAATTTCTCTGTTTC	SDM
5YdbL E60A	CATTAATGCCGCACGTAAAGCGAGTTACCAACAACTGGC	SDM
3YdbL E60A	GCTTTACGTGCGGCATTAATGTCGGCTACTAATTTCTCTG	SDM
5YdbL E60D	CATTAATGCCGACCGTAAAGCGAGTTACCAACAACTGGC	SDM
3YdbL E60D	GCTTTACGGTCGGCATTAATGTCGGCTACTAATTTCTCTG	SDM
5YdbL R61A	ATGCCGAAGCTAAAGCGAGTTACCAACAACTGGCAAAGC	SDM
3YdbL R61A	CTCGCTTTAGCTTCGGCATTAATGTCGGCTACTAATTTC	SDM
5YdbL R61K	ATGCCGAAAAGAAAGCGAGTTACCAACAACTGGCAAAG	SDM
3YdbL R61K	CTCGCTTTCTTTTCGGCATTAATGTCGGCTACTAATTTC	SDM
5YdbL Y65A	AAGCGAGTGCCCAACAACTGGCAAAGCAAAATAAGTGTC	SDM
3YdbL Y65A	CAGTTGTTGGGCACTCGCTTTACGTTCGGCATTAATGTC	SDM
5YdbL L68A	TACCAACAAGCGGCAAAGCAAAATAATGTGTCGGTAGATG	SDM
3YdbL L68A	TTGCTTTGCCGCTTGTTGGTAACTCGCTTTACGTTCGG	SDM
5YdbL N72A	CAAAGCAAGCTAATGTGTCGGTAGATGATATCGCGAAAC	SDM
3YdbL N72A	GACACATTAGCTTGCTTTGCCAGTTGTTGGTAACTCGC	SDM
5YdbL D77A	GTGTCGGTAGCTGATATCGCGAAACTCGCCGGGCAAAAG	SDM
3YdbL D77A	GCGATATCAGCTACCGACACATTATTTTGCTTTGCCAG	SDM
5YdbL I79A	GTAGATGATGCCGCGAAACTCGCCGGGCAAAAGCTCGTA G	SDM
3YdbL I79A	GAGTTTCGCGGCATCATCTACCGACACATTATTTTGCTTT G	SDM
5YdbL K86A	CCGGGCAAGCGCTCGTAGCTCGGGCCAAACCGGGGG	SDM
3YdbL K86A	GCTACGAGCGCTTGCCCGGCGAGTTTCGCGATATCATC	SDM
5YdbL L87A	GGCAAAAGGCCGTAGCTCGGGCCAAACCGGGGGAATAT G	SDM
3YdbL L87A	CGAGCTACGGCCTTTTGCCCGGCGAGTTTCGCGATATC	SDM
5YdbL R90A	CTCGTAGCTGCGGCCAAACCGGGGGAATATGTACAAGGG	SDM
3YdbL R90A	GGTTTGGCCGCAGCTACGAGCTTTTGCCCGGCGAGTTTC	SDM
5YdbL R90K	CTCGTAGCTAAGGCCAAACCGGGGGAATATGTACAAGGG	SDM
3YdbL R90K	GGTTTGGCCTTAGCTACGAGCTTTTGCCCGGCGAGTTTC	SDM
5YdbL W104A	ACGGTAAAGCGGTGCGAAAATTTTAACTCGAGCACCACC	SDM
3YdbL W104A	TTTCGCACCGCTTTACCGTTTATCCCTTGTACATATTC	SDM
5YdbL K107A	GGGTGCGAGCATTTTAACTCGAGCACCACCACCACCACC	SDM
3YdbL K107A	GAGTTAAAATGCTCGCACCCATTTACCGTTTATCCCTTG	SDM
5pBAD33YdbL deletion	AAGCTTGGCTGTTTTGGCGGATGAGAGAAGATTTTCAGCC	SDM
3pBAD33pET 2342RBS	ATGTATATCTCCTTCTTAAAGAGCTCGAATTCGCTAGCCC	SDM

5YdbH-YdbL_SacI	ATCAGAGCTCAATCAGGATATTAACTACCCATGCTGGGTAAATATAAAGCCGTTCTCGC	Cloning YdbH-YdbL into pBAD33
3YdbH-YdbL_HindIII	GCGCAAGCTTTTAAAATTTTCGCACCCATTTACCGTTTATCCCTTG	Cloning YdbH-YdbL into pBAD33
3pBAD33RBS YdbH-YdbL	GAGCTCGAATTCGCTAGCCCAAAAAAACGGGTATGGAG	SDM
3pBAD33RBS NoStart	GGGTAGTTAATATCCTGATTGAGCTCGAATTCGCTAGCCC	SDM
3pBAD33RBS Start	CATGGGTAGTTAATATCCTGATTGAGCTCGAATTCGCTAG	SDM
5pBAD33spec P1	CATAATGAAATAAGATCACTACCGGGCGTATTTTTTGAGTTATCGAGATTTTCAGGAGCTAAGGAAGCTAAAATGGCTATTTGTTTATTTTTCTAAATAC	Swapping Chloramphenicol cassette with spectinomycin cassette
3pBAD33spec P2	GCCATTCATCCGCTTATTATCACTTATTCAGGCGTAGCACCAGGCGTTTAAGGGCACCAATAACTGCCTTAAAAAAACCCCTCGGCTTGAACGAATTGTTAGAC	Swapping Chloramphenicol cassette with spectinomycin cassette
5pET2342RBSYdbH	TTTAAGAAGGAGATATACATATGCTGGGTAAATATAAAGCCGTTCTCGC	SDM
5pET2342RBSYdbL	TTTAAGAAGGAGATATACATATGAAGAAAACATTACTTCTTTGTGCGTTTCTTG	SDM
3pBAD33YdbHdeletion	ATGTATATCTCCTTCTTAAAGAGCTCGAATTCGCTAGC	SDM
5pBAD33YdbL deletion	AAGCTTGGCTGTTTTGGCGGATGAGAGAAGATTTTCAGC	SDM
5YdbH_UP	TGTGCTATAAACGGCGAG	Amplifying Δ*ydbH-ydbL::kan*
3YdbL_Down	GCCAGGCGAGTATCGAC	Amplifying Δ*ydbH-ydbL::kan*
OBFCOX005	ATTTTGTTTAACTTTGGCCCAGGAGATATACATATGCTGGGTAAATATAAAGCCGTTCTC	N-ter YdbH
OBFCOX006	AGCAGCCGGATCGGCCGGCCGGTCTCTCTTAAAATTTTCGCACCCATTTACCGTTTATCC	C-ter YdbL
YdbL_Nterm_SS_his_F	CCATCACCATCACCATGGTAGTGGAGAAAATCTGTATTTTCAGGGGACTCTGGATGAAGCCAGAACTCAG	N-ter YdbL fragment 1
T7_R	AGAGGCCCCAAGGGGTTATGCTA	N-ter YdbL tag fragment 1
YdbH_Seq4	GTTCAGCAATTAACTGGTTGCGCTATAT	N-ter YdbL tag fragment 2
YdbL_Nterm_SS_His_R	CAGATTTTCTCCACTACCATGGTGATGGTGATGGTGATGGTGATGGTGCAATGCCATTACATTGCTGCTT	N-ter YdbL tag fragment 2
pBFC052	ACTTTGGCCCAGGAGATATACATATGCATCACCACCATCACCATGAAAATCTGTATTTTCAGGGGTTGACTCTGGATGAAGCCAGAACTC	N-ter tag before YdbL
oBFC053	CTTTGTTAGCAGCCGGATCGGCCGGCCGGTCTCTCTTAAAATTTTCGCACCCATTTACCGTTTATCC	N-ter tag before YdbL

oGLI228	TAACTTTGGCCCAGGCGGCCAGGAGGGTCTCACATGAAG AAAACATTACTTCTTTGTGCG	N-ter tag before YdbL
oGLI229	CCTTAATGGTGATGGTGGTGATGGGTCTCGCCCGATTAAA ATTTTCGCACCCATTTACCG	N-ter tag before YdbL
CDX115	AATTGTGAGCGGATAACAATTCCCCTCT	SDM
CDX116	ATCTACCGACACATTATTTTGCTTTGCCGCTTGTTGGTAAC TCGCTTTACGTTCGG	SDM
CDX117	GCCGAACGTAAAGCGAGTTACCAACAAGCGGCAAAGCAA AATAATGTGTCGGTAGATGATATCG	SDM
CDX118	CAGCAGCCAACTCAGCTTCCT	SDM
CDX119	ATATTCCCCCGGTTTGGCCCGAGCTACCGCCTTTTGCCC GGCGAGTTTCG	SDM
CDX120	GATATCGCGAAACTCGCCGGGCAAAAGGCGGTAGCTCGG GCCAAACCGG	SDM
CDX121	CTTTGCCAGTTGTTGGTAACTCGCTTTCGCTTCGGCATTA ATGTCGGCTACTAATTTCTCT	SDM
CDX122	AAATTAGTAGCCGACATTAATGCCGAAGCGAAAGCGAGTT ACCAACAACTGGCAAA	SDM
CDX123	CTTTGCCAGTTGTTGGTAACTCGCTTTTTTTTCGGCATTAA TGTCGGCTACTAATTTCTCT	SDM
CDX124	AAATTAGTAGCCGACATTAATGCCGAAAAAAAAGCGAGTT ACCAACAACTGGCAAA	SDM
CDX125	TTTCGCGATATCATCTACCGACACATTCGCTTGCTTTGCC AGTTGTTGGTAACTCG	SDM
CDX126	GCGAGTTACCAACAACTGGCAAAGCAAGCGAATGTGTCG GTAGATGATATCGCGAAACT	SDM
CDX127	TGCCAGTTGTTGGTAACTCGCTTTACGCGCGGCATTAATG TCGGCTACTAATTTCTCTGTTTC	SDM
CDX128	GAGAAATTAGTAGCCGACATTAATGCCGCGCGTAAAGCG AGTTACCAACAACTGGC	SDM
CDX129	TGCCAGTTGTTGGTAACTCGCTTTACGTTTGGCATTAATGT CGGCTACTAATTTCTCTGTTTC	SDM
CDX130	GAGAAATTAGTAGCCGACATTAATGCCAAACGTAAAGCGA GTTACCAACAACTGGC	SDM
CDX131	TTCCCCCGGTTTGGCCCGAGCTACGAGCGCTTGCCCGGC GAGTTTCGC	SDM
CDX132	GATGATATCGCGAAACTCGCCGGGCAAGCGCTCGTAGCT CGGGCCAAACC	SDM
CDX133	TACGAGCTTTTGCCCGGCGAGTTTCGCCGCATCATCTACC GACACATTATTTTGCTTTGCC	SDM
CDX134	AAGCAAAATAATGTGTCGGTAGATGATGCGGCGAAACTCG CCGGGCA	SDM
CDX135	CCCTTGTACATATTCCCCCGGTTTGGCCGCAGCTACGAG CTTTTGCCCGG	SDM
CDX136	AAACTCGCCGGGCAAAAGCTCGTAGCTGCGGCCAAACCG GGGGAATATGTACAAG	SDM
CDX137	CCCTTGTACATATTCCCCCGGTTTGGCTTTAGCTACGAGC TTTTGCCCGG	SDM
CDX138	AAACTCGCCGGGCAAAAGCTCGTAGCTAAAGCCAAACCG GGGGAATATGTACAAG	SDM
CDX139	CACATTATTTTGCTTTGCCAGTTGTTGCGCACTCGCTTTAC GTTCGGCATTAATGTC	SDM
CDX140	GACATTAATGCCGAACGTAAAGCGAGTGCGCAACAACTG GCAAAGCAAAATAATGTGTCG	SDM

CDX141	TACCCGCCCCTGAGTTCTGGCTTCATCCGCAGTCCCCTGAAAATACAGATTTTCTCCACTAC	SDM
CDX142	GGAGAAAATCTGTATTTTCAGGGGACTGCGGATGAAGCCAGAACTCAGGGGC	SDM
CDX143	GTGGTGATGGGTCTCGCCCGATTAAAACGCTCGCACCCATTTACCGTTTATCCCT	SDM
CDX144	CAAGGGATAAACGGTAAATGGGTGCGAGCGTTTTAATCGGGCGAGACCCATCAC	SDM
CDX145	AAATGTTTCACCTACCCGCCCCTGAGTCGCGGCTTCATCCAGAGTCCCCTGAAAATA	SDM
CDX146	TATTTTCAGGGGACTCTGGATGAAGCCGCGACTCAGGGGCGGGTAGGTG	SDM
CDX147	GGTCTCGCCCGATTAAAATTTTCGCACCGCTTTACCGTTTATCCCTTGTACATATTCCCCC	SDM
CDX148	GAATATGTACAAGGGATAAACGGTAAAGCGGTGCGAAAATTTTAATCGGGCGAGAC	SDM
CDX158	GAAGCCAGAACTCAGGGGCGGGTAGGTGCCACATTTTACGGTTATCTGGTTGCGCTG	SDM
CDX159	CAGCGCAACCAGATAACCGTAAAATGTGGCACCTACCCGCCCCTGAGTTC	SDM
CDX160	GAAGCCAGAACTCAGGGGCGGGTAGGTGATACATTTTACGGTTATCTGGTTGCGCTG	SDM
CDX161	CAGCGCAACCAGATAACCGTAAAATGTATCACCTACCCGCCCCTGAGTTC	SDM

Table S3 - Details of plasmid construction

Plasmid name	Template used for PCR	Primers used	Purpose
pBAD33RBSYdbH/ YnbE/YdbL	pBAD33NativeYdbH/ YnbE/YdbL	5pET2342RBSYdbH 3pBAD33RBSYdbH-YdbL	To introduce strong ribosome binding site (RBS) from pET2342 plasmid upstream of YdbH
pBAD33RBSYdbH/ YnbE	pBAD33RBSYdbH/YnbE/YdbL	5pBAD33YdbLdeletion 3YdbLdeletion	To delete *ydbL* from the template plasmid
pBAD33RBSYdbH	pBAD33RBSYdbH/YnbE	5pBAD33YdbLdeletion 3YnbE deletion	To delete *ynbE* from the template plasmid
pBAD33RBSYnbE	pBAD33RBSYdbH/YnbE	5YdbH deletion 3pBAD33pET2342RBS	To delete *ydbH* from the template plasmid
pBAD33RBSYdbL	pBAD33NativeYdbL	5pET2342RBSYdbL 3pBAD33RBSYdbH-YdbL	To introduce strong ribosome binding site (RBS) from pET2342 plasmid upstream of YdbL
pET23/42GST-His_2-YdbH /YnbE/FLAG-YdbL	pET23/42GST-His_2-YdbH /YnbE/YdbL	5YdbL NtermFLAG 3YdbL NtermFLAG	To introduce C-terminal FLAG-tag in YdbL
pET23/42YdbL	pET23/42GST-His_2-YdbH/YnbE/YdbL	5YdbH-YnbE deletion 3pET2342NdeIstart	To delete *ydbH-ynbE* from the template plasmid
pET23/42FLAG-YdbL	pET23/42GST-His_2-YdbH /YnbE/FLAG-YdbL	5YdbH-YnbE deletion 3pET2342NdeIstart	To delete *ydbH-ynbE* from the template plasmid
pGLI120	BW25113 *Escherichia coli*	OBFCOX005 and OBFCOX006	To produce untagged YdbH-YdbL operon in custom pET vector
pGLI139	pGLI120	YdbL_Nterm_SS_his_F and T7_R (Fragment 1, F1) YdbH_Seq4 and YdbL_Nterm_SS_His_R (Fragment 2, F2)	To produce N-ter His_6-TEV tagged YdbL in YdbH-YdbL custom pET vector for periplasmic expression (signal sequence + 1AA before tag, 1-22)
pGLI171	BW25113 *Escherichia coli*	oBFC052 and pBFC053	To produce N-ter His_6-TEV tagged YdbL(22-108) in custom pET vector for cytoplasmic expression (without signal sequence 1-21).
pGLI190	pGLI139	oGLI228 and oGLI229	To produce N-ter His_6-TEV tagged YdbL in custom pET vector for periplasmic expression (signal sequence + 1AA before tag, 1-22)

pCD079	pGLI190	CDX115 and CDX116 (F1)<br>CDX117 and CDX118 (F2)	SDM
pCD080	pGLI190	CDX115 and CDX119 (F1)<br>CDX120 and CDX118 (F2)	SDM
pCD081	pGLI190	CDX115 and CDX121 (F1)<br>CDX122 and CDX118 (F2)	SDM
pCD082	pGLI190	CDX115 and CDX123 (F1)<br>CDX124 and CDX118 (F2)	SDM
pCD083	pGLI190	CDX115 and CDX125 (F1)<br>CDX126 and CDX118 (F2)	SDM
pCD084	pGLI190	CDX115 and CDX127 (F1)<br>CDX128 and CDX118 (F2)	SDM
pCD085	pGLI190	CDX115 and CDX129 (F1)<br>CDX130 and CDX118 (F2)	SDM
pCD086	pGLI190	CDX115 and CDX131 (F1)<br>CDX132 and CDX118 (F2)	SDM
pCD087	pGLI190	CDX115 and CDX133 (F1)<br>CDX134 and CDX118 (F2)	SDM
pCD088	pGLI190	CDX115 and CDX135 (F1)<br>CDX136 and CDX118 (F2)	SDM
pCD089	pGLI190	CDX115 and CDX137 (F1)<br>CDX138 and CDX118 (F2)	SDM
pCD090	pGLI190	CDX115 and CDX139 (F1)<br>CDX140 and CDX118 (F2)	SDM
pCD091	pGLI190	CDX115 and CDX141 (F1)<br>CDX142 and CDX118 (F2)	SDM
pCD092	pGLI190	CDX115 and CDX143 (F1)<br>CDX144 and CDX118 (F2)	SDM
pCD093	pGLI190	CDX115 and CDX145 (F1)<br>CDX146 and CDX118 (F2)	SDM
pCD094	pGLI190	CDX115 and CDX147 (F1)<br>CDX148 and CDX118 (F2)	SDM
pCD097	pGLI190	CDX115 and CDX159 (F1)<br>CDX158 and CDX118 (F2)	SDM
pCD098	pGLI190	CDX115 and CDX161 (F1)<br>CDX160 and CDX118 (F2)	SDM

Table S4 - Plasmids used in this study

Plasmid ID	Details	Use of plasmid	Source
pBE1092	Custom pET vector with N-ter His_6 tag	For cloning of expression plasmids	(4)
pDCE587	Custom pET vector with C-ter His_6 tag	For cloning of expression plasmids	(4)
pGLI120	pBE1092_YdbH-YdbL	Used as a template for pGLI139 construction	This study
pGLI139	pBE1092_YdbH_YnbE_YdbL(1-22)_His_6-TEV-YdbL(23-108)	Used as template for pGLI190 construction	This study
pGLI171	pBE1092_His_6-TEV-YdbL(22-108)	To express and purify full-length YdbL for structural studies (XRC and NMR). Signal sequence (1-21) removed for cytoplasmic expression before tag.	This study
pGLI190	pDCE587_YdbL(1-22)_His_6-TEV-YdbL(23-108)	To express and purify full-length YdbL for native MS and biochemical analyses. Signal sequence + 1AA before tag for periplasmic expression.	This study
pCD079	pDCE587_YdbL(1-22)_His_6-TEV_YdbL(23-108)_L68A	To express and purify mutant form of YdbL	This study
pCD080	pDCE587_YdbL(1-22)_His_6-TEV_YdbL(23-108)_L87A	To express and purify mutant form of YdbL	This study
pCD081	pDCE587_YdbL(1-22)_His_6-TEV_YdbL(23-108)_R61A	To express and purify mutant form of YdbL	This study
pCD082	pDCE587_YdbL(1-22)_His_6-TEV_YdbL(23-108)_R61K	To express and purify mutant form of YdbL	This study
pCD083	pDCE587_YdbL(1-22)_His_6-TEV_YdbL(23-108)_N72A	To express and purify mutant form of YdbL	This study
pCD084	pDCE587_YdbL(1-22)_His_6-TEV_YdbL(23-108)_E60A	To express and purify mutant form of YdbL	This study
pCD085	pDCE587_YdbL(1-22)_His_6-TEV_YdbL(23-108)_E60K	To express and purify mutant form of YdbL	This study
pCD086	pDCE587_YdbL(1-22)_His_6-TEV_YdbL(23-108)_K86A	To express and purify mutant form of YdbL	This study
pCD087	pDCE587_YdbL(1-22)_His_6-TEV_YdbL(23-108)_I79A	To express and purify mutant form of YdbL	This study
pCD088	pDCE587_YdbL(1-22)_His_6-TEV_YdbL(23-108)_R90A	To express and purify mutant form of YdbL	This study
pCD089	pDCE587_YdbL(1-22)_His_6-TEV_YdbL(23-108)_R90K	To express and purify mutant form of YdbL	This study
pCD090	pDCE587_YdbL(1-22)_His_6-TEV_YdbL(23-108)_Y65A	To express and purify mutant form of YdbL	This study
pCD091	pDCE587_YdbL(1-22)_His_6-TEV_YdbL(23-108)_L24A	To express and purify mutant form of YdbL	This study
pCD092	pDCE587_YdbL(1-22)_His_6-TEV_YdbL(23-108)_K107A	To express and purify mutant form of YdbL	This study
pCD093	pDCE587_YdbL(1-22)_His_6-TEV_YdbL(23-108)_R28A	To express and purify mutant form of YdbL	This study

pCD094	pDCE587_YdbL(1-22)_His$_6$-TEV_YdbL(23-108)_W104A	To express and purify mutant form of YdbL	This study
pCD097	pDCE587_YdbL(1-22)_His$_6$-TEV_YdbL(23-108)_E35A	To express and purify mutant form of YdbL	This study
pCD098	pDCE587_YdbL(1-22)_His$_6$-TEV_YdbL(23-108)_E35D	To express and purify mutant form of YdbL	This study

Table S5 - Expected versus calculated experimental masses for YdbL variants complexed with YnbE peptide by native MS.

YdbL Variant	Expected mass apo (Da)	Expected mass peptide-bound (Da)	Calculated mass peptide (Da)	Charge state	Difference. (Da)
WT-apo	9600.01	12343.07	9599.44±0.98	4-	- 0.57
WT-peptide	9600.01	12343.07	12341.89±0.63	5-	- 1.18
L68A	9600.01	12343.07	12299.76±1.03	5-	- 2.03
L87A	9558.73	12301.79	12301.09±0.22	5-	- 0.70
N72A	9557.78	12300.84	12299.92±1.53	5-	- 0.92
E60A	9542.77	12285.83	12284.64±0.57	5-	- 1.19
E60K	9599.87	12342.93	12341.46±0.32	5-	- 1.47
K86A	9543.71	12286.77	12284.45±1.11	5-	- 2.32
I79A	9558.73	12301.79	12300.03±0.15	5-	- 1.76
R90A	9515.70	12258.76	12256.66±0.67	5-	- 2.10
R90K	9572.80	12315.86	12313.89±0.36	5-	-1.97
L24A	9558.73	12301.79	12300.49±1.49	5-	- 1.30
K107A	9543.71	12286.77	12285.21±0.45	5-	- 1.56
W104A	9485.67	12228.73	12227.27±0.68	5-	- 1.46
Peptide	2743.06	-	-	-	-

Table S6 - XRC data collection and refinement statistics.

	YdbL
**Wavelength**	0.7749
**Resolution range**	53.22 - 2.12 (2.33 - 2.12)
**Space group**	I 4 2 2
**Unit cell**	63.994 63.994 95.851 90 90 90
**Total reflections**	62429 (3179)
**Unique reflections**	5872 (1374)
**Multiplicity**	10.5 (10.9)
**Completeness (%)**	98.61 (95.09)
**Mean I/sigma(I)**	4.6 (1)
**Wilson B-factor**	32.99
**R-merge**	3.711 (1.373)
**R-meas**	3.902 (1.447)
**R-pim**	1.189 (0.452)
**CC1/2**	0.861 (0.514)
**Reflections used in refinement**	5872 (1374)
**Reflections used for R-free**	510 (125)
**R-work**	0.1992 (0.2921)
**R-free**	0.2412 (0.3398)
**Number of non-hydrogen atoms**	748
**macromolecules**	687
**ligands**	0
**solvent**	61
**Protein residues**	87
**RMS(bonds)**	0.006

**RMS(angles)**	0.64
**Ramachandran favored (%)**	100.00
**Ramachandran allowed (%)**	0.00
**Ramachandran outliers (%)**	0.00
**Rotamer outliers (%)**	0.00
**Clashscore**	0.72
**Average B-factor**	39.07
**macromolecules**	38.91
**solvent**	40.91

Statistics for the highest-resolution shell are shown in parentheses.

Table S7 - SEC-SAXS experimental data after data reduction.

**Sample details**		
Sample	**Cytoplasm-expressed YdbL (XRC sample)**	**Periplasm-expressed YdbL**
Source organism	*Escherichia coli* BW25113	*Escherichia coli* BW25113
Expression system	*Escherichia coli* T7 express	*Escherichia coli* T7 express
Molecular weight (Da)	9,714	9,601
Extinction coefficient (theoretical, M^{-1} cm^{-1})	11,460	11,460
Loading concentration (mg/mL)	9.9	4.8
Buffer composition	50 mM Tris pH 8, 150 mM NaCl, 1% Glycerol	50 mM Tris pH 8, 150 mM NaCl, 1% Glycerol
**SAXS data collection parameters**		
Beamline/detector	B21 Diamond Light Source, DECTRIS EIGER 4M	B21 Diamond Light Source, DECTRIS EIGER 4M
Energy (keV)	13	13
Beam size (μm)	< 75	< 75
Detector distance (m)	3.7	3.7
q-measurement range ($Å^{-1}$)	0.0045 - 0.34	0.0045 - 0.34
Normalization	Transmitted intensity	Transmitted intensity
Exposures	20 x 3 s	20 x 3 s
Configuration (batch/SEC)	SEC	SEC
Column (flow rate, ml/min)	S75 3.2/300I precision column 0.075 mL/min	S75 3.2/300I precision column 0.075 mL/min
Experimental temperature (℃)	22	22
**Data processing/structural parameters**		
SAXS Data Reduction	DAWN (Diamond Light Source)	DAWN (Diamond Light Source)
SAXS Data Processing	ScÅtter IV	ScÅtter IV
Buffer range (frames)	424 - 456	422 - 454

Main component (frames)	18 - 2520	2 - 2149
**Guinier Analysis**		
I(0) (cm^{-1})	2.95E-2	1.29E-2
R_g (Å)	13.9	13.8
qR_g range	0.1423 - 1.2990	0.1054 - 1.2987
d_{max}	40	43
R_c	0.0	0.0
P_x	4.0	4.0
P(r) analysis		
I(0)	2.86E-2	1.24E-2
R_g (Å)	13.84	13.79
d_{max} (Å)	46	43
q-range ($Å^{-1}$)	0.0045 - 0.34	0.0045 - 0.34
Score	1.125	1.178
V_p ($Å^3$)	13011	12890

Table S8 - Dali v5 server results for YdbL model. Sorted by Z-score. Top 50 hits are shown of 1000. Blue shading depicts same significant hit with ATP-dependent Clp protease proteolytic subunit.

#	Chain	Z-score	RMSD	lali	nres	%id	Description
1	5dkp-g	4.5	2.7	47	192	9	ATP-DEPENDENT CLP PROTEASE PROTEOLYTIC SUBUNIT
2	5dkp-F	4.5	2.6	47	191	9	ATP-DEPENDENT CLP PROTEASE PROTEOLYTIC SUBUNIT
3	5dkp-h	4.5	2.7	47	191	9	ATP-DEPENDENT CLP PROTEASE PROTEOLYTIC SUBUNIT
4	3s9v-B	4.5	2.7	65	759	3	ABIETADIENE SYNTHASE, CHLOROPLASTIC
5	3p5p-A	4.4	2.6	62	745	6	TAXADIENE SYNTHASE
6	5dkp-I	4.4	3.1	48	191	10	ATP-DEPENDENT CLP PROTEASE PROTEOLYTIC SUBUNIT
7	2zl4-M	4.3	3.3	48	173	21	ATP-DEPENDENT CLP PROTEASE PROTEOLYTIC SUBUNIT
8	6o9q-A	4.3	3	63	514	5	SESQUISABINENE B SYNTHASE 1
9	6mx2-E	4.3	3.3	48	182	10	ATP-DEPENDENT CLP PROTEASE PROTEOLYTIC SUBUNIT
10	5dkp-f	4.2	2.8	47	183	9	ATP-DEPENDENT CLP PROTEASE PROTEOLYTIC SUBUNIT
11	6naq-K	4.2	2.9	47	184	9	ATP-DEPENDENT CLP PROTEASE PROTEOLYTIC SUBUNIT
12	2zl4-A	4.2	3.3	48	173	21	ATP-DEPENDENT CLP PROTEASE PROTEOLYTIC SUBUNIT
13	6naq-J	4.2	2.9	47	186	9	ATP-DEPENDENT CLP PROTEASE PROTEOLYTIC SUBUNIT
14	6l3x-E	4.2	3.2	47	177	19	ATP-DEPENDENT CLP PROTEASE PROTEOLYTIC SUBUNIT
15	6mx2-N	4.2	3.3	48	180	13	ATP-DEPENDENT CLP PROTEASE PROTEOLYTIC SUBUNIT
16	6l3x-L	4.2	3.2	47	177	19	ATP-DEPENDENT CLP PROTEASE PROTEOLYTIC SUBUNIT
17	5dkp-B	4.2	2.8	47	187	9	ATP-DEPENDENT CLP PROTEASE PROTEOLYTIC SUBUNIT
18	2zl3-A	4.2	3.2	47	173	19	ATP-DEPENDENT CLP PROTEASE PROTEOLYTIC SUBUNIT
19	6mx2-L	4.2	3.4	48	183	10	ATP-DEPENDENT CLP PROTEASE PROTEOLYTIC SUBUNIT

20	6l3x-G	4.2	3.2	47	177	19	ATP-DEPENDENT CLP PROTEASE PROTEOLYTIC SUBUNIT
21	6l40-D	4.2	3.3	48	175	21	ATP-DEPENDENT CLP PROTEASE PROTEOLYTIC SUBUNIT
22	6wrf-H	4.2	3.4	48	192	13	ATP-DEPENDENT CLP PROTEASE ATP-BINDING SUBUNIT CL
23	6l3x-H	4.2	3.2	47	177	19	ATP-DEPENDENT CLP PROTEASE PROTEOLYTIC SUBUNIT
24	5dkp-J	4.2	2.8	47	193	9	ATP-DEPENDENT CLP PROTEASE PROTEOLYTIC SUBUNIT
25	6naq-H	4.2	2.9	47	187	9	ATP-DEPENDENT CLP PROTEASE PROTEOLYTIC SUBUNIT
26	6l40-I	4.2	3.2	47	175	19	ATP-DEPENDENT CLP PROTEASE PROTEOLYTIC SUBUNIT
27	7jsr-A	4.1	2.7	54	1496	13	NAD-SPECIFIC GLUTAMATE DEHYDROGENASE
28	3sae-A	4.1	2.7	64	780	5	ALPHA-BISABOLENE SYNTHASE
29	7eko-N	4.1	2.6	47	338	6	ATP-DEPENDENT CLP PROTEASE PROTEOLYTIC SUBUNIT
30	5c90-F	4.1	3.3	48	186	21	ATP-DEPENDENT CLP PROTEASE PROTEOLYTIC SUBUNIT
31	6l3x-M	4.1	3.2	47	177	19	ATP-DEPENDENT CLP PROTEASE PROTEOLYTIC SUBUNIT
32	6wr2-K	4.1	3.4	48	192	13	ATP-DEPENDENT CLP PROTEASE ATP-BINDING SUBUNIT CL
33	6l40-G	4.1	3.3	48	175	21	ATP-DEPENDENT CLP PROTEASE PROTEOLYTIC SUBUNIT
34	7wgs-N	4.1	3.3	48	180	21	ATP-DEPENDENT CLP PROTEASE PROTEOLYTIC SUBUNIT
35	6l40-J	4.1	3.3	48	175	21	ATP-DEPENDENT CLP PROTEASE PROTEOLYTIC SUBUNIT
36	6naq-E	4.1	3	47	184	9	ATP-DEPENDENT CLP PROTEASE PROTEOLYTIC SUBUNIT
37	3mt6-Z	4.1	3.3	47	183	13	ATP-DEPENDENT CLP PROTEASE PROTEOLYTIC SUBUNIT
38	5c90-A	4.1	3.3	48	179	21	ATP-DEPENDENT CLP PROTEASE PROTEOLYTIC SUBUNIT
39	8qyf-G	4.1	3.2	47	179	17	ATP-DEPENDENT CLP PROTEASE PROTEOLYTIC SUBUNIT
40	6n80-M	4.1	3.3	48	183	21	ATP-DEPENDENT CLP PROTEASE PROTEOLYTIC SUBUNIT

41	7wid-D	4.1	3.3	48	184	21	ATP-DEPENDENT CLP PROTEASE PROTEOLYTIC SUBUNIT
42	5c90-K	4.1	3.3	48	186	21	ATP-DEPENDENT CLP PROTEASE PROTEOLYTIC SUBUNIT
43	5c90-N	4.1	3.3	48	181	21	ATP-DEPENDENT CLP PROTEASE PROTEOLYTIC SUBUNIT
44	6l3x-C	4.1	3.2	47	177	19	ATP-DEPENDENT CLP PROTEASE PROTEOLYTIC SUBUNIT
45	6nb1-J	4.1	3.3	47	180	13	ATP-DEPENDENT CLP PROTEASE PROTEOLYTIC SUBUNIT
46	8qyf-E	4.1	3.2	47	179	17	ATP-DEPENDENT CLP PROTEASE PROTEOLYTIC SUBUNIT
47	7xbz-B	4.1	3.3	48	187	21	ATP-DEPENDENT CLP PROTEASE PROTEOLYTIC SUBUNIT
48	3sdv-A	4.1	4.9	63	775	6	ALPHA-BISABOLENE SYNTHASE
49	6sfx-L	4.1	3.2	48	194	17	ATP-DEPENDENT CLP PROTEASE PROTEOLYTIC SUBUNIT
50	3p2l-E	4.1	3.4	48	182	15	ATP-DEPENDENT CLP PROTEASE PROTEOLYTIC SUBUNIT

Table S9 - Numerical amide backbone chemical shift changes ordered by distance of shift (ppm).

Residue	Amide shift between apo-YdbL and peptide-bound YdbL (ppm)
L68	0.54654
L87	0.48898
R61	0.48451
N72	0.4069
E60	0.37511
A83	0.34482
K70	0.3023
L24	0.28617
A80	0.28498
I79	0.28368
Q66	0.27459
G84	0.2717
A69	0.26827
S64	0.23965
K62	0.22736
Q71	0.22698
V74	0.22613
R90	0.22132
Y65	0.21789
T29	0.20932
K81	0.19643
A89	0.18748
I57	0.18483
A59	0.16876
G34	0.16114
L82	0.15266
V97	0.14737
L41	0.14318
Y96	0.13412
Q85	0.1216
N73	0.11659
V54	0.1132

N58	0.09846
S75	0.09804
Y38	0.09524
T36	0.09417
E26	0.093
E35	0.09283
F37	0.09275
K86	0.09002
V33	0.0892
V42	0.08761
K92	0.0855
V76	0.08472
A63	0.08405
G99	0.07971
T23	0.07931
Y40	0.07679
D56	0.07551
Q67	0.07475
V88	0.07337
D78	0.07218
D25	0.06903
R32	0.06719
A91	0.0666
F108	0.06479
D77	0.05592
L44	0.05531
G39	0.05524
A27	0.05345
A43	0.05334
G94	0.05285
G31	0.04869
A55	0.04702
E51	0.04639
K107	0.04445
I100	0.04265

G102	0.04184
E95	0.04146
T46	0.03985
Q30	0.03175
V105	0.03096
N101	0.02656
R28	0.02359
T50	0.02176
R106	0.02089
K52	0.01752
K103	0.01717
W104	0.01554
Q98	0.01434
K45	0.01391
A48	0.0134
D47	0.01054
L53	0.00803
E49	0.00387

SI References

1. Blattner FR, Plunkett G, Bloch CA, Perna NT, Burland V, Riley M, Collado-Vides J, Glasner JD, Rode CK, Mayhew GF, Gregor J, Davis NW, Kirkpatrick HA, Goeden MA, Rose DJ, Mau B, Shao Y. 1997. The complete genome sequence of Escherichia coli K-12. Science 277:1453–1462.

2. Ruiz N, Davis RM, Kumar S. 2021. YhdP, TamB, and YdbH Are Redundant but Essential for Growth and Lipid Homeostasis of the Gram-Negative Outer Membrane. mBio 12:e02714-21.

3. Kumar S, Davis RM, Ruiz N. 2024. YdbH and YnbE form an intermembrane bridge to maintain lipid homeostasis in the outer membrane of Escherichia coli. Proceedings of the National Academy of Sciences 121:e2321512121.

4. Ekiert DC, Bhabha G, Isom GL, Greenan G, Ovchinnikov S, Henderson IR, Cox JS, Vale RD. 2017. Architectures of Lipid Transport Systems for the Bacterial Outer Membrane. Cell 169:273-285.e17.
